# The Cdc14 phosphatase, Clp1, does not affect genome expression

**DOI:** 10.17912/micropub.biology.001089

**Published:** 2024-02-12

**Authors:** Luis Lopez Maury, Liping Ren, Shaimaa Hassan, Jürg Bähler, Kathleen L. Gould

**Affiliations:** 1 Department of Genetics, Evolution, and Environment, Institute of Healthy Ageing, University College London, London, UK; 2 Current: Instituto de Bioquimica Vegetal y Fotosintesis, Universidad de Sevilla, Sevilla, Spain; 3 Department of Cell and Developmental Biology, Vanderbilt University School of Medicine, Nashville, TN, USA

## Abstract

*Schizosaccharomyces pombe* Clp1 is a Cdc14-family phosphatase that reverses mitotic Cdk1 phosphorylation. Despite evolutionary conservation, Clp1 ’s mammalian orthologs do not share this function. Rather, higher eukaryotic Cdc14 enzymes act in DNA repair, ciliogenesis, and gene regulation. To examine if Clp1 regulates gene expression, we compared the transcriptional profiles of cells lacking Clp1 function to that of wildtype. Because *clp1∆* cells are sensitive to the actin depolymerizing drug, LatrunculinA, we also investigated whether a transcriptional response was involved. Our results indicate that Clp1 does not detectably affect gene expression and highlight the organism-specific functions of this conserved phosphatase family.

**Figure 1. Mutations in clp1 have only marginal or no effects on the transcriptome.**
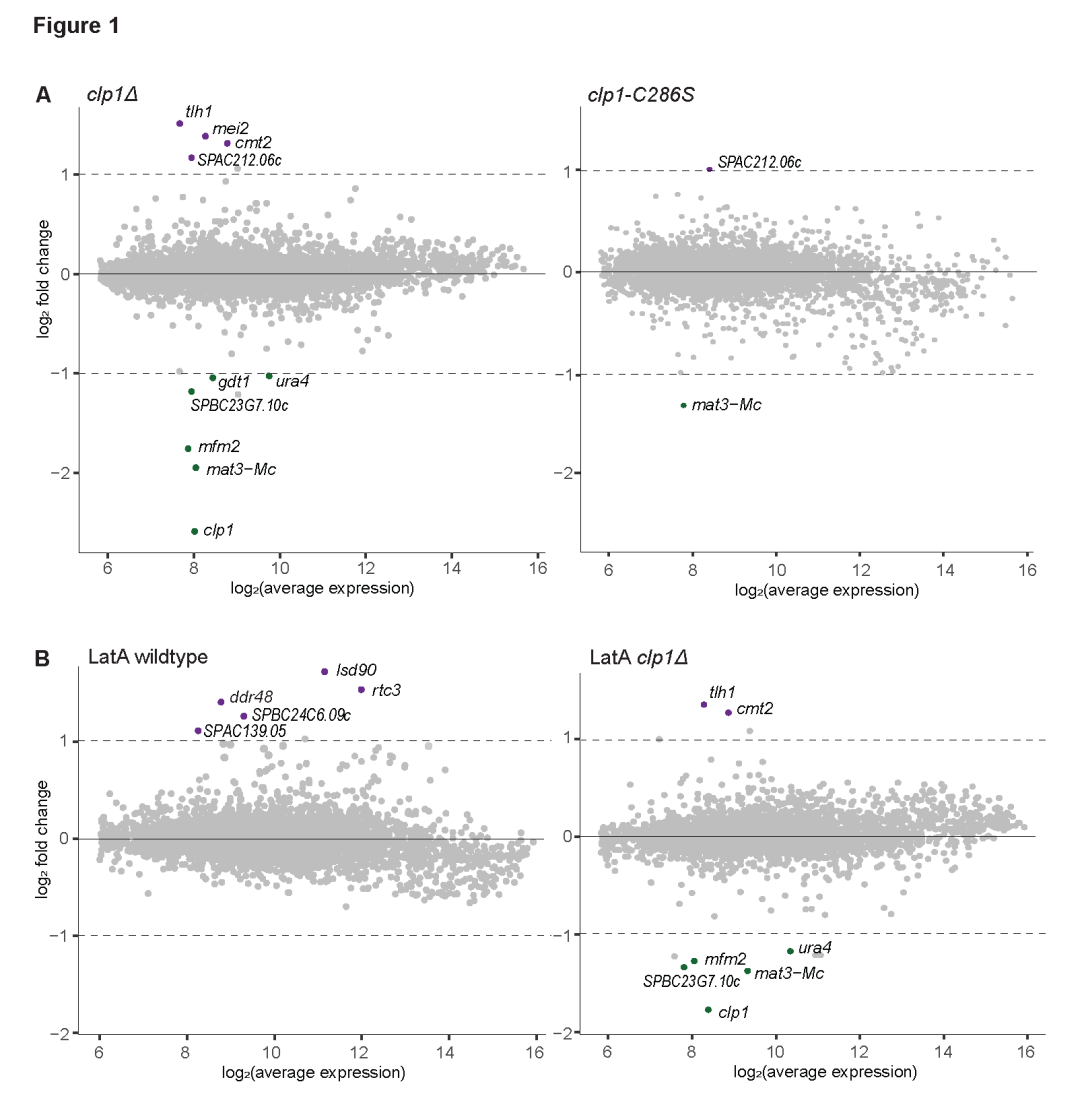
(A) MA plots of microarray expression data for *clp1Δ* relative to wildtype cells (left) and *clp1-C286S* relative to wildtype cells (right). The plotted data reflect the average of two biological repeats each, with induced (purple) and repressed (green) genes highlighted, based on a relaxed cutoff (>1.5-fold change in both repeats and >2-fold change in average of the two repeats). Names are indicated for genes that passed the cutoff. (B) MA plots of microarray expression data for LatA-treated wildtype cells relative to untreated wildtype cells (left) and LatA-treated *clp1Δ* cells relative to untreated *clp1Δ* cells (right). Visualization and cutoffs as in (A).

## Description

Cdc14 family phosphatases are highly conserved from yeast to humans (reviewed in (Mocciaro and Schiebel, 2010; Partscht and Schiebel, 2023; Stegmeier and Amon, 2004) ). These phosphatases are proline-directed with a strong preference for phosphoserines (Bremmer et al., 2012; Gray et al., 2003) and in yeast, they reverse the phosphorylation of mitotic cyclin-dependent kinase 1 ( Cdk1 ) substrates making them key regulators of mitotic exit (reviewed in (Clifford et al., 2008a; Mocciaro and Schiebel, 2010; Stegmeier and Amon, 2004) ). In higher eukaryotes, however, roles for the Cdc14 paralogs Cdc14A and Cdc14B in mitotic progression and cell proliferation have not been identified (Berdougo et al., 2008; Mocciaro et al., 2010) . Instead, these enzymes have been implicated in DNA repair and ciliogenesis (Clement et al., 2011; Clement et al., 2012; Imtiaz et al., 2018; Partscht et al., 2021; Uddin et al., 2019) ; a function in DNA repair and recombination was also found for Cdc14 in *Saccharomyces cerevisiae* (Alonso-Ramos et al., 2021; Eissler et al., 2014; Garcia-Luis et al., 2014) . Further, recent studies indicate key roles for Cdc14B in gene expression control at both the transcriptional and translational levels in several cellular processes (Dietachmayr et al., 2020; Partscht and Schiebel, 2023; Villarroya-Beltri et al., 2023) .

The sole Cdc14 enzyme in the fission yeast *Schizosaccharomyces pombe,* Clp1 (also called Flp1), reverses Cdk1 -mediated mitotic phosphorylation to support mitotic exit and cytokinesis but is not an essential protein (Chen et al., 2013; Clifford et al., 2008b; Cueille et al., 2001; Esteban et al., 2004; Fu et al., 2009; Mangione et al., 2021; Mishra et al., 2004; Trautmann et al., 2004; Trautmann et al., 2001; Wolfe and Gould, 2004) . Interestingly, like higher eukaryotic Cdc14B enzymes, Clp1 has been implicated in transcriptional regulation, specifically of cell cycle genes during the M-G1 transition (Papadopoulou et al., 2010) and in the transcriptional response to oxidative stress (Canete et al., 2023) . Here, we compared the gene transcriptional profiles of wildtype cells with cells lacking Clp1 function to determine the extent of Clp1 ’s role in *S. pombe* transcriptional regulation.

Total RNA was extracted from asynchronously growing wildtype, *clp1::ura4 ^+^* ( *clp1Δ* ) *,* and *clp1-C286S* cells. Clp1-C286S lacks catalytic activity but binds substrates (Chen et al., 2013; Denu et al., 1996; Wolfe and Gould, 2004) . RNAs were then labeled and processed for microarray hybridization in two biological repeats each for both *clp1* mutants. As expected, the *clp1* mRNA signal was much lower in *clp1Δ* cells compared to wildtype cells. Besides *clp1,* there were no substantial changes in gene expression in cells with compromised Clp1 function, even when applying a relatively relaxed cutoff ( Figure 1A ). We conclude that Clp1 plays no major, if any, role in gene regulation.

Clp1 is necessary to stabilize the cytokinetic ring when cells are treated with low doses of Latrunculin A (LatA), a compound which at higher doses in wildtype cells causes actin depolymerization (Ayscough et al., 1997; Mishra et al., 2004) . To determine if a transcriptional response was involved in the sensitivity of *clp1Δ* cells to LatA, we compared the RNA expression signatures of wildtype and *clp1Δ* cells treated with a low dose of LatA for 30 minutes, as above. Compared to untreated *clp1Δ* cells, we found no additional differences between the two strains ( Figure 1B ). These findings further indicate that Clp1 ’s role in cytokinesis does not involve changes to gene expression. We conclude that the only significant function of Clp1 in fission yeast cells is in cell cycle progression.

## Methods

Yeast and RNA preparation

*S. pombe* strains were grown in yeast extract (YE) at 32˚C (Moreno et al., 1991) and total RNA was extracted as described (Bahler and Wise, 2017) . For certain experiments, LatA (Molecular Probes, Eugene, OR, USA) in DMSO (final concentration of 0.2 µM LatA) or only DMSO was added for 30 minutes at 32˚C. KGY8378 was obtained by crossing KGY3381 ( *clp1::ura4 ^+^* *ura4-D18 ade6-M210 leu1-32 h ^-^ )* (Trautmann et al., 2001) to KGY602 ( *ura4-D18 h ^+^* ) and selecting Leu+Ade+Ura+ cells. KGY8779 was obtained by crossing KGY4972 ( *clp1-C286S* *ura4-D18 ade6-M210 leu1-32 h ^-^ )* to 975 *h ^+^* , selecting semi-wee Leu+Ade+Ura+ cells, and confirming the *clp1* mutation was present via amplifying the clp1 locus and DNA sequencing. PCR was used to determine mating types.

Microarray analysis

We used DNA microarrays displaying probes for >99% of all known and predicted genes of *S* . *pombe* spotted in duplicate onto glass slides. RNA extraction, hybridization and initial data processing and normalization were performed as previously described (Lyne et al., 2003) . Two independent biological experiments were performed each for *clp1Δ* and for *clp1-C286S* experiments as well as for LatA-treatment wildtype and *clp1Δ* experiments, including one dye swap in each case. Cut-off values of >1.5-fold change in both repeats and >2-fold change in the average of the two repeats were used for each experiment. Gene annotations were downloaded from PomBase (Harris et al., 2022) . The MA plots were prepared using the limma Bioconductor package, and the data were processed with a custom R script. ControlType weights were set to 0, spots with background channel more than 50 above the test channel were set to the median background intensity, background correction was performed with limma::backgroundCorrect() using the ‘normexp' method and an offset of 50, limma::normalizeWithinArrays() with the ‘loess' method, and limma::normalizeBetweenArrays() with the ‘Aquantile' method. The raw data from the RNA-seq experiments have been deposited in GEO repository with accession number GSE255124.

## Reagents

The strains used in this study and their genotypes are listed below.

**Strain** **Genotype** **Source**

972 *wildtype h ^-^* Lab stock

KGY8378 *clp1::ura4 ^+^ ura4-D18 h ^-^* This study

KGY8779 *clp1-C286S h ^-^* This study
